# Pelvic floor muscle training in women with urinary incontinence and pelvic organ prolapse: A protocol study

**DOI:** 10.1371/journal.pone.0308701

**Published:** 2024-08-16

**Authors:** Maria Letícia A. S. de Carvalho, Lívia Oliveira Bezerra, Joyce Maria Pereira Oliveira, Maria Clara Eugênia Oliveira, Maria T. A. B. C. Micussi

**Affiliations:** 1 Physical Therapy Department, Federal University of Rio Grande do Norte, Natal, RN, Brazil; 2 Graduate Program in Physical Therapy, Federal University of Rio Grande do Norte, Natal, Brazil; Iran University of Medical Sciences, ISLAMIC REPUBLIC OF IRAN

## Abstract

**Objective:**

To evaluate the effectiveness of pelvic floor muscle training (PFMT) on pelvic floor muscle (PFM) function and quality of life (QoL) in women with stress urinary incontinence (SUI) and pelvic organ prolapse (POP).

**Methods:**

This study will be a randomized, controlled, parallel, and blinded clinical trial. The final sample will consist of 32 women diagnosed with SUI and cystocele (stage I and II). All volunteers will be assessed and reassessed using the same protocol: assessment form, gynecological examination, functional evaluation of PFM, and questionnaires to assess quality of life, urinary function, and sexual function. All volunteers will be evaluated for satisfaction levels post-treatment. The intervention will be PFMT, totaling 16 sessions to be conducted twice a week. Reevaluation will take place at the end of treatment and 1 month after completion of PFMT. Descriptive analysis and repeated measures ANOVA will be used for result analysis. A significance level of p<0.05 will be considered for all statistical tests.

**Ethics and dissemination:**

This study has been submitted to the Ethics in Research Committee of the Federal University of Rio Grande do Norte and approved under protocol number 5.826.563. It has been registered with the Brazilian Clinical Trials Registry ReBec (RBR-49p6g3t). It is expected that these studies will provide a deeper understanding of the efficacy of PFMT in women with SUI and cystocele. Additionally, it aims to provide more insights into the efficacy of PFMT prior to surgery.

## Introduction

Among the various forms of urinary incontinence (UI), stress urinary incontinence (SUI), characterized by the involuntary loss of urine during activities that increase intra-abdominal pressure [1], is the most prevalent, affecting 25% to 45% of women, depending on the population [2]. SUI can occur in conjunction with pelvic organ prolapse (POP). Around 55% of women with stage II POP report urinary loss [3, 4]. POP´s complex origin involves risk factors like pregnancy, childbirth, connective tissue issues, aging, and PFM weakness, among others [5–8].

The anterior vaginal compartment POP, also known as cystocele, is the most frequent type of POP [9–11]. Women diagnosed with cystocele may experience various symptoms in the pelvic floor region, including a sensation of pelvic heaviness, vaginal protrusion, difficulty with urination, and sexual dysfunction [10–13]. A study [14] demonstrated that women with cystocele and/or the absence of the urethrovesical sulcus were 2.5 times more likely to experience UI.

Several studies have already established the negative impacts of both POP and UI on various aspects of women’s lives, encompassing social, occupational, domestic, psychological, physical, and sexual well-being [15–17]. Pelvic Floor Muscle Training (PFMT) has garnered Level A evidence for improving the symptoms of SUI [18, 19], enabling conscious and effective contractions [19–21] which yield positive benefits in terms of pelvic floor strength, function, and quality of life (QoL) [18, 19].

However, the clinical outcomes and cost-effectiveness relationship of PFMT in POP management remains unclear. A systematic review conducted by Cochrane [13] identified only one study involving elderly women engaged in ground exercises and health education. The review concluded that while some evidence suggests ground exercises may be beneficial for certain types of prolapse, randomized controlled trials are needed to evaluate the effectiveness of PFMT for POP treatment.

Currently, studies evaluating the efficacy of PFMT in women with genital prolapse encompass various types of POP [22–31]. A single study [32] explored PFMT for cystocele. However, the intersection of SUI and cystocele within PFMT remains unexplored. Considering that PFMT is the preferred initial treatment for incontinent women [19, 20], due to its non-invasive nature, cost-effectiveness, and minimal complications compared to surgery [14, 33], the hypothesis arises that women with SUI and cystocele could benefit from PFMT. Thus, this study aims to evaluate the effectiveness of PFMT in enhancing PFM function and the QoL in women with SUI and cystocele.

## Methodology

### Study design and research site

A controlled, randomized, parallel, and blinded clinical trial will be conducted at the Maternidade Escola Januário Cicco (MEJC) in Natal, Rio Grande do Norte, Brazil. This study has been registered with the Brazilian Clinical Trials Registry ReBec (RBR-49p6g3t) and received approval from UFRN’s Research Ethics Committee in December 19, 2022 (protocol 5,826,563). Data will be stored in the Department of Physiotherapy at the Universidade Federal do Rio Grande do Norte (UFRN) and will be archived with the principal investigator for 5 years. After this period, the data will be discarded.

### Eligibility criteria

Women with stress urinary incontinence (SUI), identified using the International Consultation on Incontinence Questionnaire—Short Form (ICIQ-SF), and a diagnosis of cystocele at stage I and II according to the Pelvic Organ Prolapse Quantification System (POP-Q) scale [34, 35] will be recruited. The participants’ age range will be between 18 and 65 years, not in menopause, and without a history of gynecological surgeries related to SUI, POP, or hysterectomy. Patients lacking cognitive capacity to comprehend and respond to the questionnaires as determined by the assessor, those with vaginal bleeding, current diagnosis of urinary tract infection, neoplasms, and/or those who decide not to continue participating in the study or withdraw their consent will be excluded.

### Ethics and disclosure

The participants will be fully informed about objectives, procedures, and voluntary participation, in accordance with Resolution No. 466/12 and the Declaration of Helsinki. Participants’ consent will be obtained through signed informed consent forms. Confidentiality of personal information is ensured throughout and after the study.

The database will be provided as part of the manuscript and its supporting information will be deposited in the journal’s repository. In addition to the database, statistical data, including means, medians, and measures of variance, will also be available.

### Participant schedule

**Fig 1:**
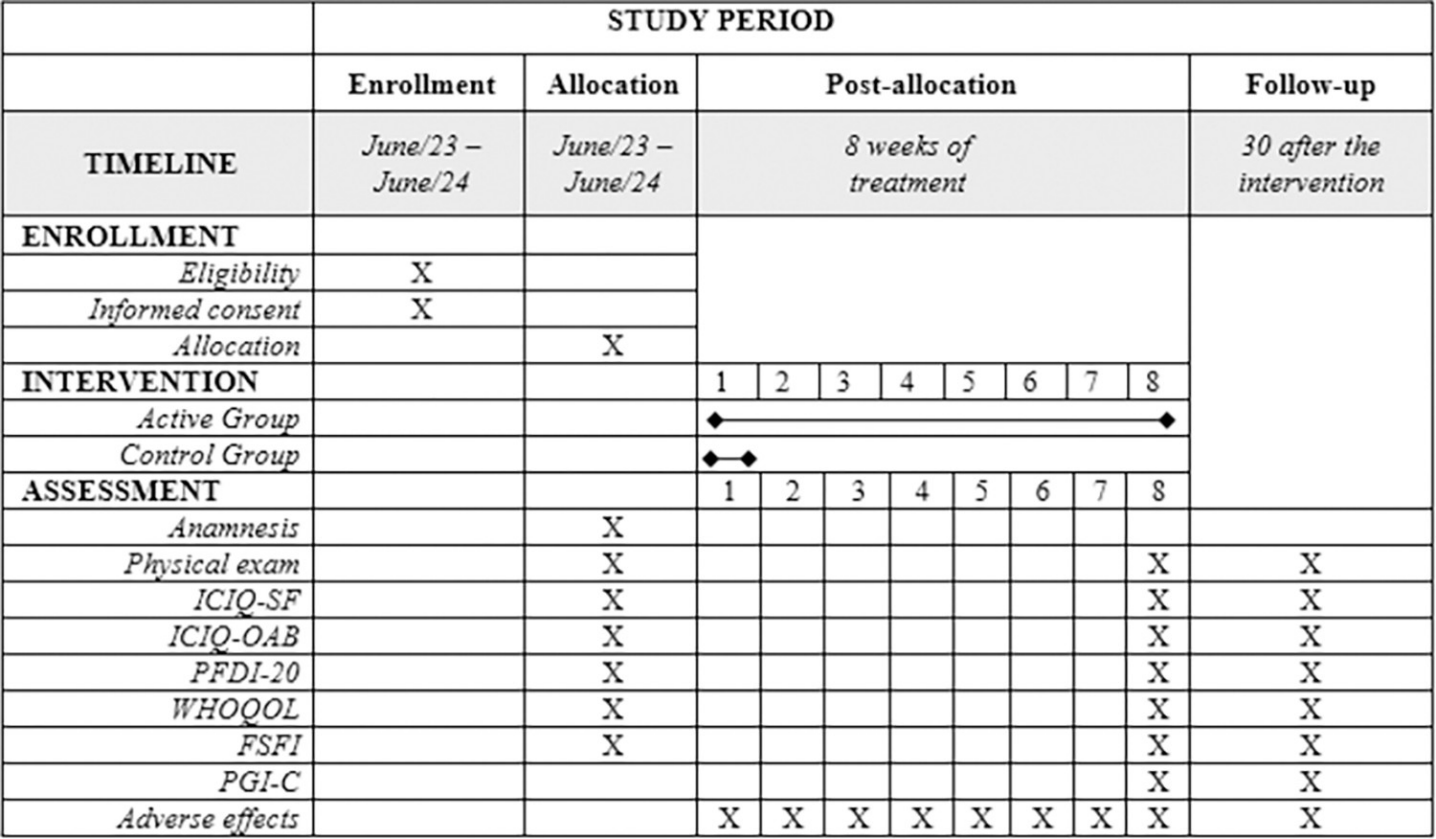
Participant schedule. **Note:** ICIQ-SF: International Consultation on Incontinence Questionnaire—Short Form; ICIQ-OAB: International Consultation on Incontinence Questionnaire Overactive; PFDI-20: Pelvic Floor Distress Inventory; WHOQOL: World Health Organization Quality of Life; FSFI: Function Sexual Female Index; PGI-C: Patient Global Impression of Change.

The participant schedule is described in Fig 1.

### Interventions

The intervention will involve individualized care, randomly divided into two groups: pelvic floor muscle training group (PFMTG) and comparator group (CG). The CG will receive informational content only, provided by a healthcare professional with expertise in urogynecology during a single visit. The educational material will encompass informative content about urinary incontinence, its risk factors, pelvic floor anatomy and function, types of UI, bladder and bowel functioning information, along with the significance of healthy lifestyle habits.

The PFMTG will receive similar educational material as the CG and will undergo 16 sessions conducted twice a week, totaling 2 months of treatment. The treatment for this group will encompass four exercise modalities: warm-up, breathing exercises, abdominal exercises, and pelvic mobility exercises. All exercises will involve pelvic floor muscle (PFM) activation. Progressions will be incorporated into each modality throughout the training.

In general, the breathing, abdominal, and pelvic mobility exercises will consist of two sets of eight repetitions during the first four weeks, progressing to three sets of eight repetitions in the final four weeks, except for the transversus contraction and plank exercises.

The PFMTG intervention will have an average duration of 45 minutes per session, with the initial five minutes dedicated to warm-up, 30 minutes for exercise execution, and approximately 10 minutes allocated to intervals between exercises.

#### Warm-up

**Fig 2:**
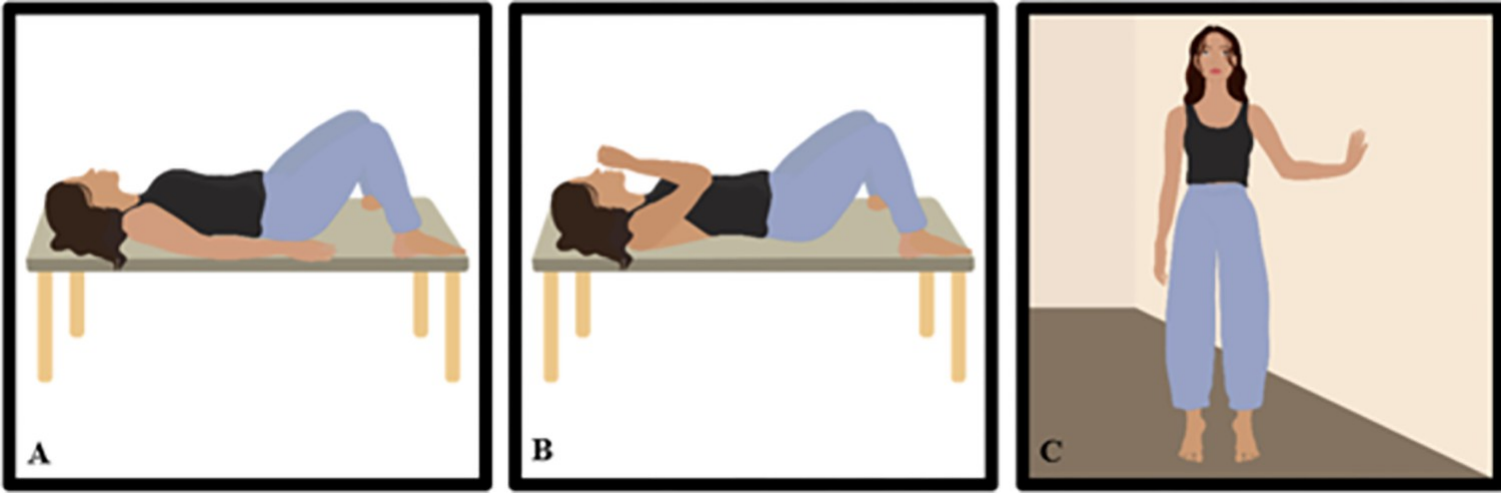
Warm-up exercises. (A) Position for quick and slow contractions of the pelvic floor muscles; (B) Cough associated with contraction of the pelvic floor muscles; (C) Bipodal position with external hip rotation and plantar flexion, involving the contraction of the pelvic floor muscles. Illustration by Luiza de Araújo.

During the initial eight sessions, participants will perform five quick contractions and five sustained slow contractions of the PFM for 3 seconds, three cough simulations associated with PFM contraction, and eight plantar flexions also linked with PFM contraction (Fig 2). In the subsequent eight sessions, the number of quick and slow PFM contractions will remain the same, while cough simulations will increase to five, and plantar flexions will be raised to 12. The rest period between each exercise will be 1 minute.

#### Breathing exercises

**Fig 3:**
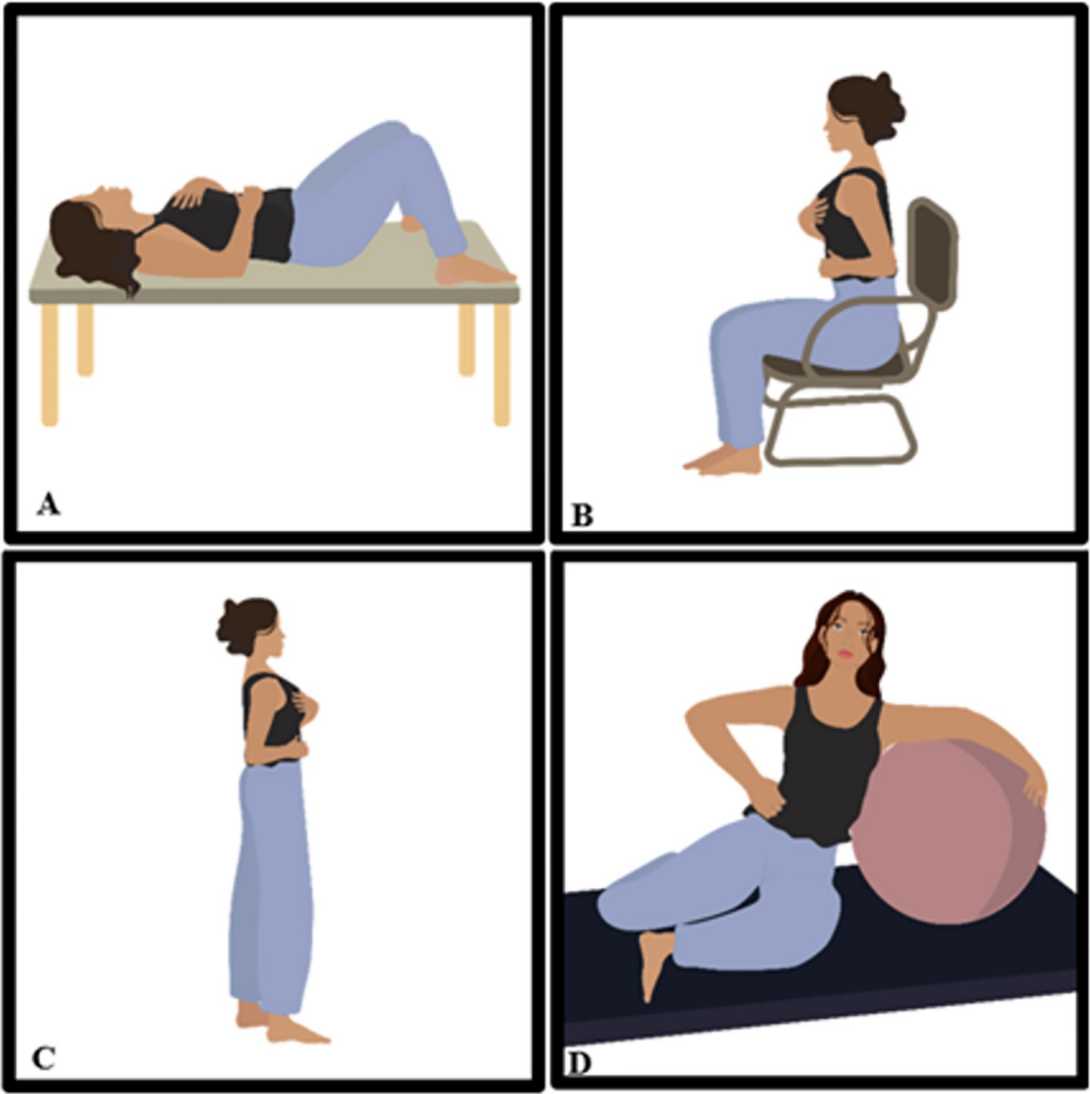
Progression of diaphragmatic breathing exercises associated with pelvic floor muscle contraction. (A) Supine position; (B) Sitting position; (C) Standing position; (D) Sitting position with the assistance of a Swiss ball, the upper limb on the same side as the ball positioned over it in a hugging manner, and the hand of the contralateral limb resting on the lateral region of the ribs on that side.

Participants will perform the diaphragmatic breathing exercises associated with PFM contractions at the time of expiration. The progression of this modality will happen by changing the postures in which is executed, as shown in Fig 3. The participant will progress in this modality every four sessions.

#### Abdominal exercises

*a) Transversus contraction*. During the initial four sessions, transversus contraction will be performed in a supine position, with the instruction to "draw the navel towards the spine" along with forced exhalation.

**Fig 4:**
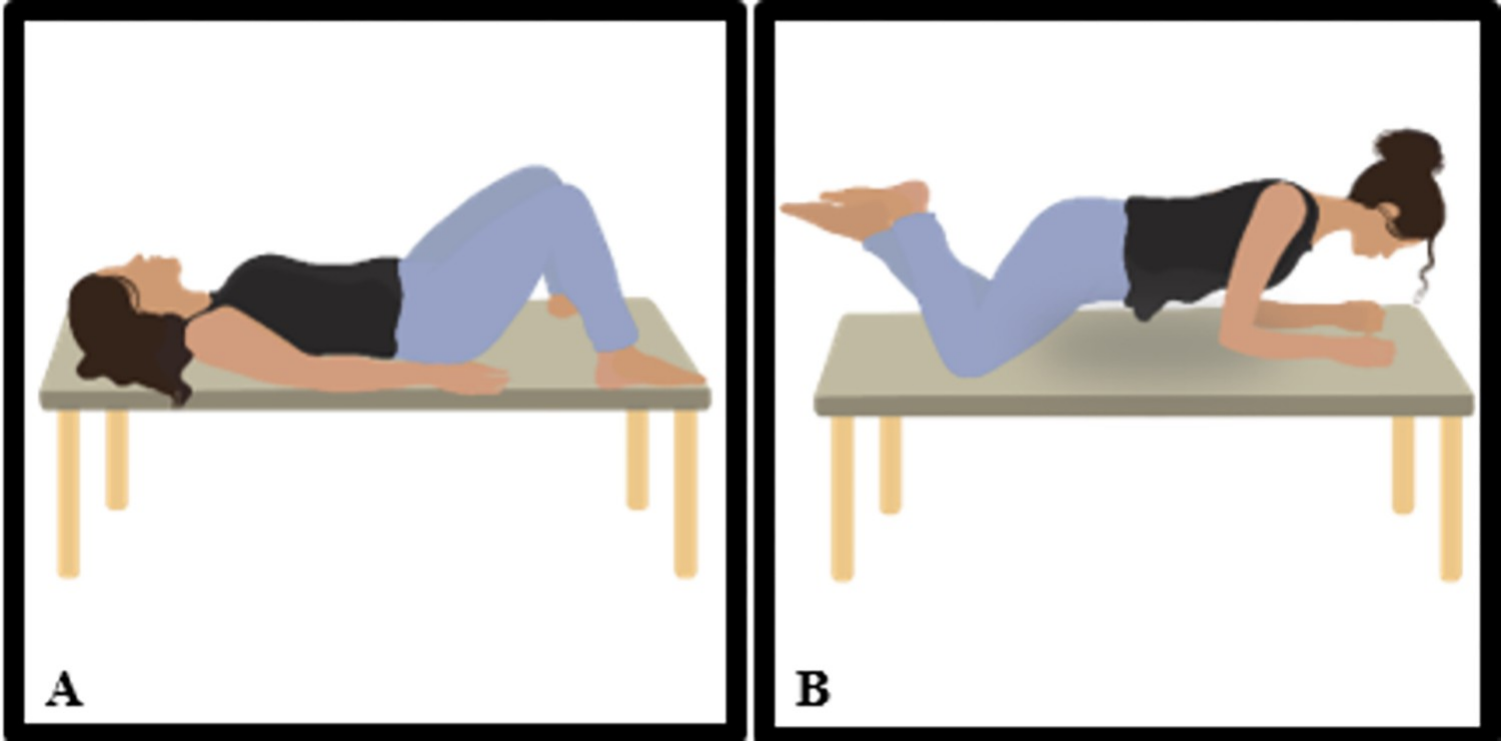
Progression of transverse muscle contraction exercises associated with pelvic floor muscle contraction. (A) Supine position; (B) Plank position. Illustration by Luiza de Araújo.

In the final 12 sessions, the participant will perform a prone plank position on the support of knees and elbows, as show in Fig 4. Initially, the participant will hold the position for five seconds, with an additional five seconds to each subsequent session, ending with 60 seconds on the support. PFM contraction will be prompted every five seconds during the plank. Thus, in the first session, the participant will perform one contraction, and by the end, 12 contractions will be completed.

**Fig 5:**
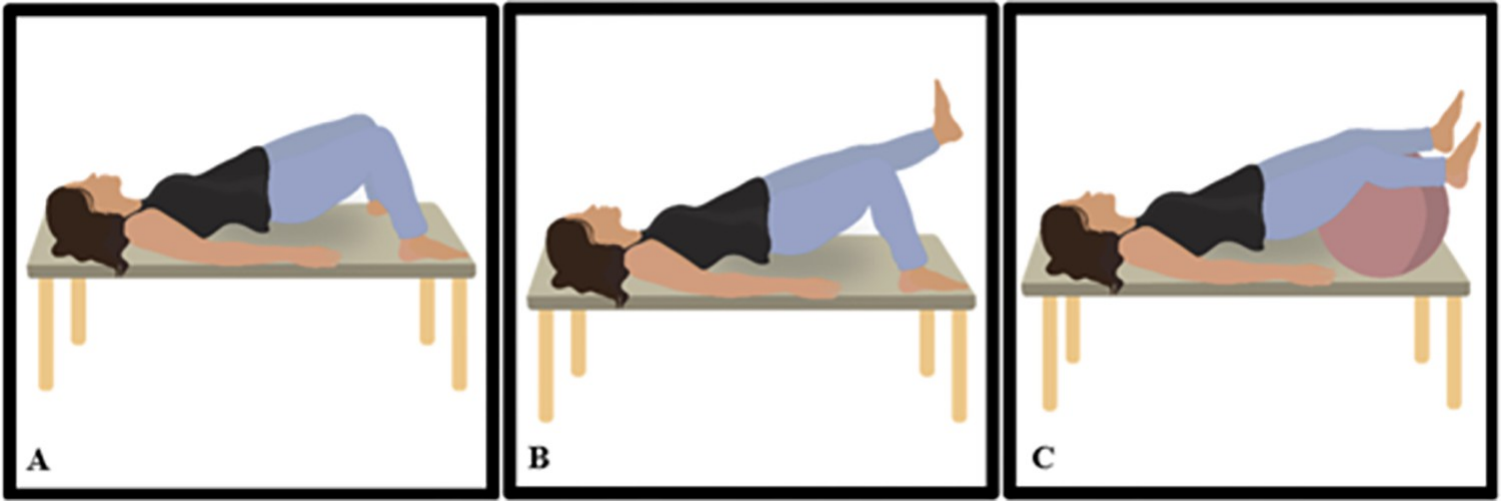
Progression of bridge exercises associated with pelvic floor muscle contraction. (A) Bridge position with bipodal support; (B) Bridge position with unipodal support; (C) Bridge position with bilateral support of lower limbs on the Swiss ball at calf height. Illustration by Luiza de Araújo.

*b) Bridge*. Bridge exercise will be conducted in a supine position with support on the mat. The PFM contraction will occur at the start of the hip elevation movement and continue until they return to the initial position. Progression will occur through position changes (Fig 5).

#### Pelvic mobility exercises

**Fig 6:**
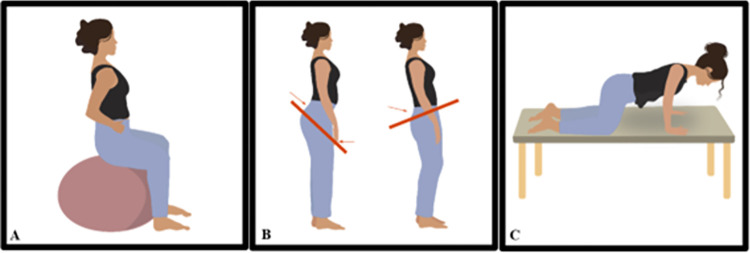
Progression of pelvic mobility exercises associated with pelvic floor muscle contractions. (A) Seated position on the Swiss ball; (B) Standing position; (C) Four-point kneeling position. Illustration by Luiza de Araújo.

Pelvic mobility exercises will be performed in the directions of anterior and posterior tilt, with the PFM contraction prompted during the posterior tilt. Progression will be achieved through position changes (Fig 6).

### Results

**Fig 7:**
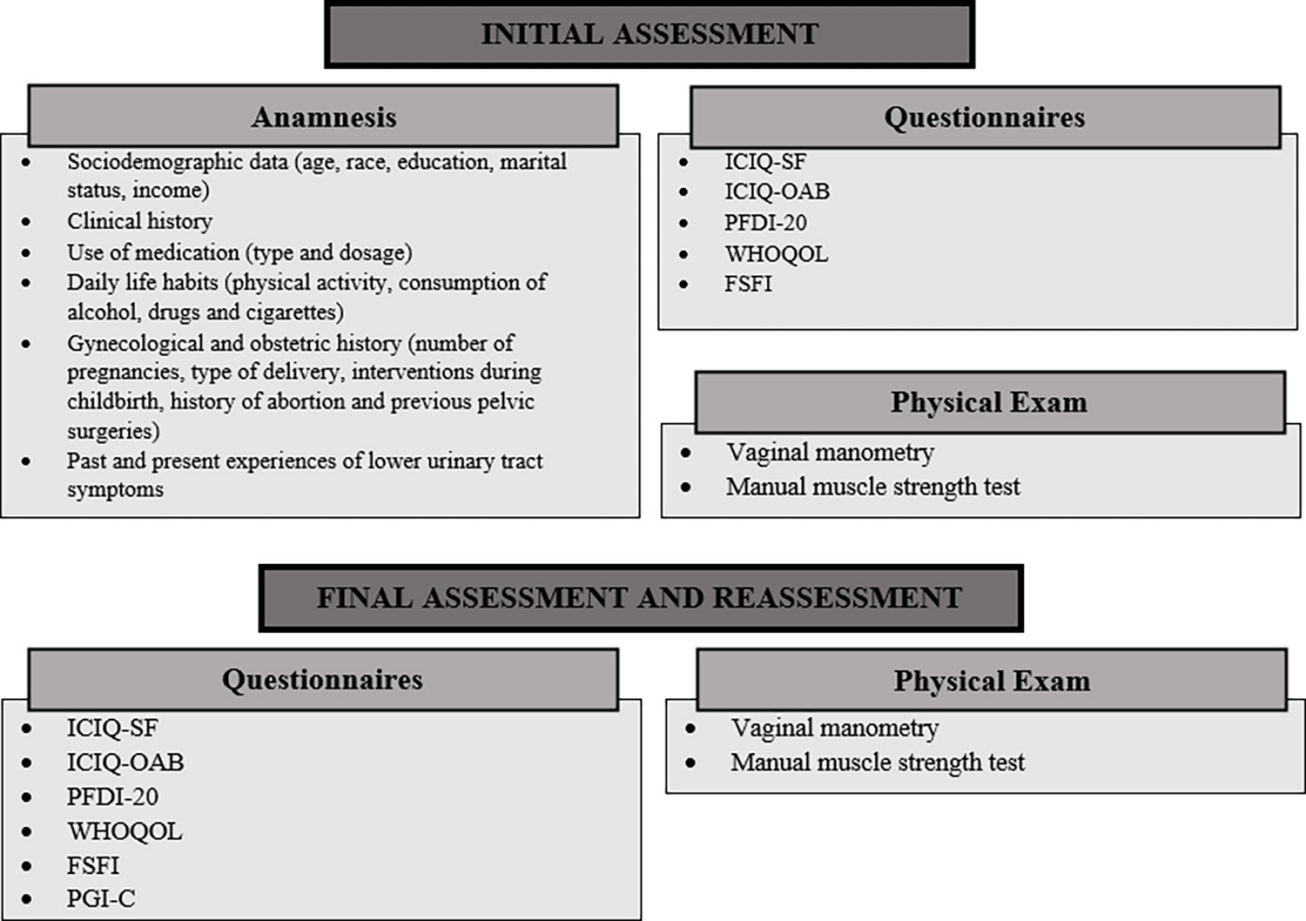
Assessment and re-assessment items. **Note:** ICIQ-SF—International Consultation on Incontinence Questionnaire; ICIQ-OAB—International Consultation on Incontinence Questionnaire Overactive; PFDI -20—Pelvic Floor Distress Inventory; WHOQOL-bref—World Health Organization Quality of Life; FSFI—Function Sexual Female Index; PGI-C—Patient Global Impression of Change.

All assessments will be conducted by a blinded evaluator. The volunteers will undergo initial evaluation, including medical history, gynecological examination, functional assessment of the PFM, and questionnaires. The assessments will take place at three time points: before treatment (baseline assessment), at the end (final assessment), and one month after the last session (re-assessment). Fig 7 illustrates the assessment and re-assessment items.

The primary outcome will be PFM pressure measured by vaginal manometry. Secondary outcomes will include manual muscle strength testing (Modified Oxford Scale) and questionnaires: International Consultation on Incontinence Questionnaire—Short Form (ICIQ-SF), International Consultation on Incontinence Questionnaire Overactive (ICIQ-OAB), Pelvic Floor Distress Inventory (PFDI-20), World Health Organization Quality of Life (WHOQOL), Female Sexual Function Index (FSFI), and Patient Global Impression of Change (PGI-C).

#### Vaginal manometry

The pressure exerted by the PFM will be measured using the PeritronTM model 9300AV, achieved through a probe inserted into the vaginal canal. The value is given in cmH2O, thus representing an indirect measure of muscle strength [36]. For the examination, the volunteer will assume the lithotomy position. The volunteer will be instructed to perform three maximal contractions, with a 30-second interval between them [37], and instructed not to associate the PFM contraction with abdominal muscles, hip adductors, or glutes. The considered value will be the average of the three attempts and categorized based on pressure as very weak (7.5–14.5), weak (14.6–26.5), moderate (26.6–41.5), good (41.6–60.5), and strong (>60.6) [38].

#### Manual muscle strength testing

The evaluator performs bidigital palpation and instructs the volunteer to contract the PFM with the command "squeeze my finger with maximum force you can." It is necessary for the volunteer not to associate PFM contraction with accessory muscles. Muscle strength is graded according to the Modified Oxford Scale [39], which classifies muscle strength into six grades, from grade 0 to 5.

#### International Consultation on Incontinence Questionnaire—Short Form (ICIQ-SF)

ICIQ-SF consists of four questions assessing the frequency, severity, and impact of UI, along with a set of eight self-diagnostic items that evaluate the causes or situations of UI experienced by patients. Only the first three questions are scored, and the total score ranges from zero to twenty-one points. The impact of UI on QoL is divided into: no impact (0 points); mild impact (1 to 3 points); moderate (4 to 6 points); severe (7 to 9 points), and very severe (10 or more points) [40].

#### International Consultation on Incontinence Questionnaire Overactive (ICIQ-OAB)

ICIQ-OAB was developed to specifically assess urinary symptoms related to Overactive Bladder Syndrome (OAB), through four questions related to frequency, nocturia, urgency, and incontinence. The ICIQ-OAB generates an overall score ranging from 0 to 16 points, reflecting the impact on the patient’s QoL. Each question includes a scale ranging from 0 (no discomfort) to 10 (very uncomfortable) to measure the level of symptom discomfort [41].

#### Pelvic Floor Distress Inventory (PFDI-20)

PFDI has 20 questions divided into 3 domains (bladder/intestine/pelvis) in their sub-scales: Pelvic Organ Prolapse Distress Inventory (POPDI-6), Colorectal-Anal Distress Inventory (CRADI-8), and Urinary Distress Inventory (UDI6). The first question raised is the presence or absence of the symptom described in each question. If the response is positive, there is quantification of the symptom, where the patient has options: "not at all"/"a little"/"moderately"/"quite a bit". The response options are quantified from zero (no symptoms) to 4 (significant impact of the symptom). The average of the total scores per sub-scale is multiplied by 25 and finally, the three scores are summed [42].

#### World Health Organization Quality of Life (WHOQOL)

The WHOQOL questionnaire assesses four domains to evaluate quality of life (QoL): physical, psychological, social relationships, and environment. Responses are rated on a Likert-type scale, ranging from 1 to 5, where higher scores indicate a higher QoL. To calculate the final score, responses from each domain are summed and divided by the number of questions within that domain, resulting in the following classification: needs improvement (1 to 2.9); fair (3 to 3.9); good (4 to 4.9); very good (5) [43].

#### Female Sexual Function Index (FSFI)

FSFI has 19 questions grouped into six domains: desire, arousal, lubrication, orgasm, satisfaction, and pain. All questions are multiple-choice, and each answer is assigned a value from 0 to 5. Scores are calculated by a mathematical formula, resulting in a sexual function score ranging from 2 to 36 points, with a cutoff point of 26.5 points. A lower score indicates poorer sexual function, and a score equal to or below this point indicates sexual dysfunction [44].

#### Patient Global Impression of Change (PGI-C)

The PGI-C is a 7-item scale ranging from "1 = no change" to "7 = very much better," where the patient can broadly evaluate their improvement throughout the treatment, involving personal and psychometric aspects inherent to the treatment process [45].

### Sample size

The sample size was pre-determined using G-Power 3.1.9.2 (F-tests—ANOVA: repeated measures, between groups), based on a previous study [46] utilizing vaginal manometry data. The sample size was estimated with a significance level of 0.05, power of 98%, and effect size of 0.23. According to this methodology, the sample size resulted in 27 participants. An additional 20% for possible losses (5 volunteers) was added. Thus, 32 volunteers will be recruited and randomized into two groups of 16 participants each.

### Recruitment

It will be a randomized, controlled, parallel, and blinded clinical trial, with recruitment of volunteers from June 01, 2023 to June 30, 2024. The sample will consist of users from the general gynecology outpatient clinic of the MEJC who present SUI and a diagnosis of stage I or II cystocele.

### Randomization, allocation concealment, and blinding

The randomization process will be carried out electronically through the website http://www.randomization.com to assign each participant to the PFMT or CG. Allocation will be 1:1, and each participant will have an equal probability of belonging to either group. Two pieces of information will be entered: the sample size and the number of groups. Website will generate specific coding for each group and will distribute subjects randomly. To ensure blinding in this study, the processes of randomization, assessment/reassessment, training of selected volunteers, and data analysis will be performed by distinct researchers.

### Data collection

Data will be collected at the MEJC, Brazil.

### Statistical analysis

Data will be analyzed using the Statistical Package for Social Science (SPSS) version 22.0, and will be used Excel Software version 2019 to capture the data. Initial analysis will present sociodemographic and clinical parameters in descriptive tables showing mean values, standard deviation, frequency, medians, and percentiles. Additionally, 95% confidence intervals will be presented. To assess initial group differences, unpaired t-test or Mann-Whitney test will be used for continuous variables, and chi-square test for categorical variables. Depending on data normality, repeated measures ANOVA or Kruskal-Wallis test followed by Tukey post-test will identify differences. Time-group interaction and intra-group variations for studied variables will be analyzed. Mauchly test will check sphericity, and if violated, Greenhouse-Geisser correction will be applied. Effect size (Cohen’s f2) and statistical power will also be calculated. Significance level of 5% will be considered.

### Data monitoring

This study will not involve participants at risk of death or harmful interventions; therefore, there will be no need to create a data monitoring committee.

### Adverse effects

Adverse effects of PFMT are rare. Any discomfort or pain linked to treatment will be assessed. Volunteers will be closely monitored, asked about session discomfort, and adverse events documented. If risks arise, interventions halt. Withdrawn participants still factor into intention-to-treat analysis.

## Supporting information

S1 Checklist Spirit checklist. (DOCX)
